# Sepsis-driven atrial fibrillation and ischaemic stroke. Is there enough evidence to recommend anticoagulation?

**DOI:** 10.1016/j.ejim.2021.10.022

**Published:** 2022-04

**Authors:** Isuru Induruwa, Eleanor Hennebry, James Hennebry, Mrinal Thakur, Elizabeth A. Warburton, Kayvan Khadjooi

**Affiliations:** a Department of Stroke, Cambridge University Hospitals, Cambridge CB2 0QQ, United Kingdom; b Department of Clinical Neurosciences, University of Cambridge, Cambridge CB2 0QQ, United Kingdom; c Department of Medicine, Royal Sussex County Hospital, Brighton BN2 5BE, United Kingdom

**Keywords:** Sepsis, Infection, Atrial Fibrillation, Ischaemic Stroke, Stroke prevention, Anticoagulation

## Abstract

Sepsis can lead to cardiac arrhythmias, of which the most common is atrial fibrillation (AF). Sepsis is associated with up to a six-fold higher risk of developing AF, where it occurs most commonly in the first 3 days of hospital admission. In many patients, AF detected during sepsis is the first documented episode of AF, either as an unmasking of sub-clinical AF or as a newly developed arrhythmia. In the short term, sepsis that is complicated by AF leads to longer hospital stays and an increased risk of inpatient mortality. Sepsis-driven AF can also increase an individual's risk of inpatient stroke by nearly 3-fold, compared to sepsis patients without AF. In the long-term, it is estimated that up to 50% of patients have recurrent episodes of AF within 1-year of their episode of sepsis. The common perception that once the precipitating illness is treated or sinus rhythm is restored the risk of stroke is removed is incorrect. For clinicians, there is a paucity of evidence on how to reduce an individual's risk of stroke after developing AF during sepsis, including whether to start anticoagulation. This is pertinent when considering that more patients are surviving episodes of sepsis and are left with post-sepsis sequalae such as AF. This review provides a summary on the literature available surrounding sepsis-driven AF, focusing on AF recurrence and ischaemic stroke risk. Using this, pragmatic advice to clinicians on how to better detect and reduce an individual's stroke risk after developing AF during sepsis is discussed.

## Highlights

• Sepsis can lead to cardiac arrhythmias such as atrial fibrillation (AF).
• It is estimated that up to 50% of patients have recurrent episodes of AF within 1-year of their episode of sepsis.
• These patients are at increased risk of ischaemic stroke; three-fold during their inpatient stay and two-fold up to at least 4 years from the initial episode of sepsis.
• There are no guidelines on how to prevent stroke in these patients, therefore, many do not receive appropriate treatment to alleviate their ischaemic stroke risk.

## 1 Introduction

Atrial fibrillation (AF) is the most commonly encountered arrhythmia in clinical practice and is associated with significant morbidity and mortality [1]. Currently, it is estimated that between 2 and 4% of adults have AF [2]. AF increases the risk of ischaemic stroke five-fold [3] due to cardiac thromboembolism and in a number of patients admitted with ischaemic stroke, the first diagnosis of AF is at the time of their stroke, due to its subclinical nature [4]. Anticoagulation is indicated for stroke prevention in AF, either with warfarin or a direct oral anticoagulant (DOAC), in patients with a score ≥ 1 in men, or ≥ 2 in women [5,6] as calculated by the CHA_2_DS_2_-VASc risk assessment tool.

Stroke prevention in patients with first diagnosed AF in the context of critical illness, post-surgery, trauma, electrolyte abnormalities, excess alcohol intake, thyrotoxicosis, pregnancy and sepsis is an important yet unresolved clinical dilemma. Even though this type of ‘secondary’ AF is often driven by a reversible cause, it is unclear whether AF persists intermittently after treatment of the precipitating condition, thereby increasing the risk of subsequent stroke. For example, transient post-operative AF, which occurs in 5–30% of patients after non-cardiac surgery, with a peak incidence in the first four weeks carries a four- to five-fold risk of recurrent AF in the next 5 years [6]. For this reason, it has been recognised as a risk factor for ischaemic stroke, other thrombotic diseases and death by the European Society of Cardiology [6].

AF, first diagnosed in the setting of sepsis accounts for approximately 20% of AF driven by an acute precipitant [7]. Due to a lack of complete understanding of its pathophysiology, a paucity of defining studies and clear guidelines, commencing anticoagulation in such patients remains a point of contention. A recent UK-wide survey indicated that 64% of physicians do not routinely anticoagulate, and 68% do not routinely use stroke risk-assessment scores, in critically ill patients with new-onset AF [8].

This review provides an overview of sepsis-driven AF, its outcomes with respect to AF recurrence, ischaemic stroke risk and mortality, and discusses a pragmatic approach to managing stroke-risk in this patient cohort.

## 2 Methods

We conducted a systematic search to retrieve articles pertaining to first-diagnosed AF in patients with an infection or sepsis. We selected research papers with a range of different study designs and methods amongst those identified by a PubMed and EMBASE search using index terms: ‘infection, sepsis, stroke, atrial fibrillation’, combined as search sets using Boolean operators (AND, OR). Within the studies discovered, we focused on those covering first diagnosed AF prevalence, risk of recurrence, risk of ischaemic stroke and death, outside of the post-operative setting. From the research papers identified, six studies were of particular interest as they described stroke-outcomes of first-diagnosed AF during sepsis (Table 1).

**Table 1:** Summary of atrial fibrillation recurrence, ischaemic stroke and mortality outcomes of first-diagnosed atrial fibrillation in patients with sepsis.

Outcomes with sepsis and first-diagnosed AF.									
Author	Type of study	Setting	Sepsis and first diagnosed AF (*n*)	Incidence of first diagnosed AF in sepsis (%)	% AF recurrence (time)	Incidence of in-hospital stroke (%)	Risk of In hospital stroke	Long term stroke risk (time)	% Mortality (time)
Salman et al.	Retrospective cohort		21	25.9	NC	0	NC	NC	72 (28-day)
Walkey et al. [9]	Retrospective review from database in USA	Hospitalised adults with severe sepsis	2896	5.9	NC	2.6%	*OR= 2.70 (2.05–3.57, *P* <0.001)	NC	56.3(in hospital)
Walkey et al. [39]	Retrospective review from database in USA	Hospitalised adults with severe sepsis	9540	6.9	44.2 (1 year) 54.9 (5 years)	NC	NC	*HR 1.22 (1.10–1.36, *P* <0.001) (5 years)	46.2 (1 year) 74.8 (5 year)
Cheng et al. [19]	Retrospective review from database in Taiwan	Septicaemia survivors	1286	1.9	NC	NC	NC	*OR 1.74 (1.26–2.41) (before 1 year)	NC
Cheng et al. [10]	Retrospective review from database in Taiwan	Septicaemia survivors	182	2.5	NC	NC	NC	HR 1.88 (1.37–2.65) (mean 4.5 years)	NC
Gundlund et al. [40]	Retrospective review from database in Denmark	Hospitalised adults with an infection	30,307	2.2	36 (1 year)	NC	NC	HR 1.94 (1.85–2.05) (1 year)^£^	HR 1.61(1.57–1.65) (1 year)

AF = atrial fibrillation, OR = odds ratio, HR = hazard ratio, NC = not calculated, ND = not disclosed, ns = non-significant.

*Multivariable adjusted ^£^Included all thromboembolic events (ischaemic stroke, transient ischaemic attack and arterial embolus).

## 3 Sepsis and atrial fibrillation

Hospitalised patients with sepsis demonstrate an increased risk of developing first diagnosed AF compared to those without sepsis, with sepsis being associated with up to a six-fold higher risk of developing AF [9]. The prevalence of sepsis-driven AF is quoted to be between 2 and 26% [9], [10], [11], which increases to over 40% if the patient is in septic shock [12,13] and is detectable in 44% of patients undergoing inpatient 7-day Holter monitors [14]. Commonly, infections arising from the respiratory tract, urinary tract or an abdominal source appear to be most associated with AF occurence [15], [16], [17]. Recent work also suggest that AF developed in patients hospitalised with coronavirus disease 2019 (COVID-19) also carries high risk of mortality and stroke, although further research in this condition is needed to make firm conclusions [18]. What is known is that patients who develop AF during sepsis tend to be older [19] and are more likely to have other cardiovascular comorbidities [19] and therefore a higher CHA_2_DS_2_-VASc score. AF can develop anytime during the presentation with sepsis, but most commonly documented between days 1–3 admission [12]. In a study of 1087 AF episodes in 418 patients admitted to the intensive care unit (ICU), the median duration of their first AF episode was 5 h (inter quartile range (IQR), 2–11), compared with 4 h (IQR, 2–10) for a recurrent episode (*P* = 0.001) [13]. This is important as the 2020 ESC guidelines suggest that a time documented AF of greater than 30 s is diagnostic, and warrants consideration of anticoagulation [6]. However, the pathophysiology of what drives AF is as multifactorial as the disease itself and various factors have been described in the literature to be instrumental in AF driven by sepsis.

### 3.1 Inflammation

In a non-critical setting, there are many theories for the pathogenesis of AF, particularly a model where there are multiple re-entrant circuits, increased automaticity in various areas of the atria or a combination of both which leads to uncontrolled, disorganised electrical activity [20]. Prior to even developing sepsis, the presence of vascular risk factors, such as hypertension, diabetes and congestive cardiac failure, in the setting of a low-level systemic inflammation, causes electrical and atrial remodelling, perpetuating the arrhythmia.

Sepsis is also fundamentally an inflammatory condition, with well-established increases in the production of inflammatory mediators; cytokines, coagulation factors, free radicals, and vasoactive intermediates; a major part of the inflammatory response [21]. It is this inflammatory response that is likely to be responsible for the development, or the unmasking of, subclinical AF in those with sepsis and relevant risk factors. For example, Meirehenrich et al. demonstrated a significant rise in *C*-reactive protein (CRP) level prior to the onset of sepsis-induced AF [22] and Klouwenberg et al. ascertained that patients who developed sepsis-driven AF had a higher CRP compared to those who did not (*P* < 0.0001) [13] intimating a close relationship between sepsis, inflammation and AF.

### 3.2 Organ dysfunction

Whether AF first diagnosed in the context of sepsis is a marker of general organ dysfunction, or whether the AF itself drives or contributes to organ dysfunction is not clear. Bosch et al., in their meta-analysis looking at risk factors for new AF in sepsis, suggested that any organ dysfunction, but particularly acute cardiac, respiratory and renal failures are more closely correlated with AF development, compared to other organs [23,24].

In the context of hypovolaemia related to septic shock, the use of intravenous fluids, with the aim of higher mean arterial pressures causing atrial stretch was associated with a higher incidence AF [25]. Similarly, the use of vasopressors such as noradrenaline and dobutamine to correct hypotension was also correlated with a higher incidence of AF [26] which could be through mechanisms such as increased myocyte automaticity [27].

### 3.3 Electrolyte imbalances

Critically ill patients, especially with sepsis, are well documented to be at high risk of electrolyte disturbances through a variety of pathophysiological mechanisms [28]. This in turn can lead to the manifestation of cardiac arrhythmias such as AF with disturbances in sodium, potassium, magnesium and phosphate. For example, in ICU admissions with sepsis, both hyponatraemia and hypokalaemia are commonly observed. Such changes in sodium and potassium levels can cause sinoatrial node dysfunction and pulmonary vein depolarisation, both of which can contribute to the development of AF [29], [30], [31].

In the atria and ventricles, magnesium modulates potassium and calcium channels and therefore, has been shown to have stabilising properties within the atrium [32]. However, magnesium alterations, especially hypomagnesaemia, are common during sepsis [33] and can lead to both ventricular and atrial arrhythmias. Studies have documented associations between hypomagnesaemia and AF [34] and the rate of AF genesis has been shown to be related to the severity of hypomagnesaemia [35] hence its usage in controlling atrial arrhythmias, especially in ICU [36]. Similarly, there is a high prevalence of hypophosphatemia in septic patients, associated with the development of new cardiac arrhythmias, including AF [37].

## 4 Recurrence of sepsis-driven AF after the initial episode

When considering sepsis-driven AF, or AF driven by any cause, the common perception is that once the cause is treated, AF and the stroke risk is eliminated. However, this may not be the case. In Lubitz et al.’s study of 1400 patients with first-diagnosed AF, 7% (*n* = 102) of secondary AF was caused by acute infection [38]. 53% of those with infection-driven AF had recurrence, and although the authors do not specify when, 59% AF recurrences in the whole cohort occurred within 2.5 years of the first episode. They also concluded that, patients with infection-driven AF had over a 1.6-fold increase in developing recurrent AF (adjusted HR 1.64 (1.00–2.7), *P* = 0.05) compared to other causes of secondary AF. Walkey et al. identified first diagnosed AF in 9540 patients hospitalised with sepsis. They discovered that the risk of AF occurrence post discharge is 44% at 1-year and 55% at 5-years in those who had first diagnosed AF compared to 7.7% and 15.5%, respectively, in those without AF during sepsis (*P* < 0.0001*)* [39]. In a cohort of 10,000 participants, Wang et al. recently demonstrated that the risk of recurrent AF in the setting of sepsis is lower than of AF without a precipitant, but that AF did recur and that it was associated with significant long-term morbidity and mortality [16]. Finally, in 30,000 Danish patients hospitalised with an infection who developed new AF, 36% of those had AF when readmitted to hospital with AF within 1-year, although the true incidence may even be higher if we consider the patients who would have developed AF, but were not readmitted to hospital [40]. In the same study, the authors concluded that those with infection-related AF were more likely to remain in AF, compared to those who did not develop AF during their infection (adjusted HR 25.98 (95% CI 24.64–27.39). As mentioned earlier, a possible explanation here is the unmasking of previous subclinical AF in the context of sepsis and inflammation, or the presence of an atrial susceptibility or AF substrate in these patient groups which increases the risk of developing AF.

## 5 Outcomes

### 5.1 Non-stroke outcomes

In the short term, sepsis that is complicated by AF leads to a longer hospital stay and an increased risk in mortality [7]. In a cohort of 21 patients that developed new AF in the setting of sepsis, the 28-day mortality was significantly higher (*P* = 0.04) compared to those who did not develop AF, with an increased predicted mortality rate (OR 1.020 (95%CI 1.001–1.038, *P* = 0.03) at 28-days [11]. Wang et al. discovered that regardless of the presence of an initial precipitant, recurrent AF was associated with increased adjusted risks of heart failure (HR 2.74 [95% CI, 2.39–3.15]; *P* < 0.001) and mortality (HR 2.96 [95% CI, 2.70–3.24]; *P* < 0.001) [16]. Similarly, Walkey et al. reported a cumulative incidence of heart failure of 11% and mortality of 75% at 5-years, in patients with first diagnosed AF during sepsis, significantly higher than in those without AF (*P* < 0.0001*)*. Other studies, including a systematic review by Gandhi et al., have demonstrated a similar significant rise in risk of in-hospital mortality if sepsis is complicated by AF suggesting that sepsis-related AF has significant adverse effects outside of just ischaemic stroke [41].

### 5.2 In-hospital and short-term stroke outcomes with sepsis-driven AF

One important question is whether newly diagnosed sepsis-driven AF is more thrombogenic than already known AF? It could well be that in the presence of sepsis and thus systemic inflammation and hypercoagulation, the immediate risk of stroke is increased. This is highlighted by work of Walkey et al., who showed that in patients with sepsis, in-hospital ischaemic stroke occurred in 2.6% of individuals with sepsis-driven transient AF compared to 0.57% of those with pre-existing AF and were at greater inpatient stroke risk compared to sepsis patients without AF (adjusted OR = 2.70 (2.05–3.57, *P* < 0.001) [9]. This subsequently increased the risk of inpatient mortality in these cohorts. Cheng et al. reported that middle-aged sepsis survivors were most at risk of ischaemic stroke from AF, within 3 months of the episode of sepsis [19] and also at a mean follow-up time of 4.5 years, compared to those who were AF free, HR ໿3.56 (95% CI 1.32–9.63) [10] highlighting that even patients without classic stroke risk-factors may also be at risk of ischaemic stroke after developing sepsis-driven AF.

### 5.3 Long term stroke outcomes with sepsis-driven AF

There is also evidence that the long-term risk of ischaemic stroke goes well beyond the initial episode of sepsis and AF. Lubitz et al. demonstrated that the long-term risks of incident stroke is similar whether the AF was driven by a precipitant or not [38]. Walkey et al. showed that the adjusted risk of ischaemic stroke at 5-years was significantly higher in those with new sepsis-driven AF, compared to those without AF (HR 1.22 (95% CI 1.1–1.36), *P* < 0.0001) [42]. Similarly, Cheng et al. reported an increase in long-term stroke risk of 74% (OR 1.74 (1.26–2.41) before1-year) [19] and (HR 1.88 (1.37–2.65) (mean 4.6 years)) [10] in patients with sepsis-driven AF. Gundlund et al. recently reported a cumulative incidence of thromboembolic events (ischaemic stroke, TIA or arterial embolism) of 7.6% for those with infection-related AF and 4.4% for those with infection without AF, at 1-year, showing an increased risk of thromboembolic events with infection-related AF (adjusted HR 1.91, 95% CI 1.81–2.02) [40].

These results are particularly pertinent as they highlight an increased risk of stroke in patients with AF post sepsis, compared to those who did not have AF. It is therefore becoming increasingly clear that as patients recover from sepsis, especially as survival rates for sepsis improve, a strategy to manage those at risk of post-sepsis cardioembolic stroke needs to be formulated.

## 6 Is there enough evidence to anticoagulate?

Should we consider anticoagulation in all patients who develop sepsis-driven AF? The studies so far demonstrate a high probability of AF recurrence, increased risk of non-stroke morbidity, ischaemic stroke in the short- and long-term, as well as death. There are limitations to the available data, given the lack of randomised data and the retrospective nature of the studies that describe ischaemic stroke risk in sepsis-driven AF. Nevertheless, one study reports that nearly half the patients who have an ischaemic stroke after a hospitalisation for sepsis and new AF, do not receive another diagnosis of AF prior to their stroke [42]. Furthermore, the risk of ischaemic stroke in sepsis-driven AF is higher than in those without AF during sepsis, as shown in Xiao et al's meta-analysis of the studies comparing stroke outcomes of first diagnosed AF compared to no AF, with a pooled OR for stroke of 1.88 (95% CI 1.13–3.14, *P*<0.05) [43]. Sepsis, therefore, must be recognised as a driver of AF and linked with the development of acute thrombi.

Further evidence can be gleaned from looking at stroke-risk caused by other similar AF-triggers, for example non-cardiac surgery, causing transient episodes of AF. A recent systematic review and meta-analysis concluded that patients who developed post-operative AF following non-cardiac surgery carried a four-fold higher risk of stroke and a 3.5-fold higher risk of all-cause mortality compared to those who did not develop AF post-operatively [44]. However, in this cohort, whether anticoagulation leads to a reduction in thromboembolic events in these patients remains to be fully evaluated [45].

In the absence of guidelines, a pragmatic way of approaching stroke prevention in sepsis-driven AF is to divide treatment strategies into acute and sub-acute stages. There are only a few studies that have looked at outcomes in AF patients who have been anticoagulated during the acute period of sepsis. The most robust argument for caution when anticoagulating in the acute stage of sepsis is from Walkey and colleagues’ analysis of outcomes of 35,500 patients after in-hospital anticoagulation for sepsis-induced AF [17]. 35.3% received anticoagulants but rates of inpatient ischaemic stroke did not significantly differ (RR 0.94; 95% CI, 0.77–1.15). Clinically significant bleeding occurred more often amongst anticoagulated patients (RR, 1.21; 95% CI, 1.10–1.32). They could find no consistent evidence that the potentially increased risk of bleeding with anticoagulation during sepsis was offset by lower rates of in-hospital stroke. Quon et al. reported no clear association between oral anticoagulation and a lower incidence of ischaemic stroke (OR: 1.98 [95% CI: 0.29–13.47), but conversely no significant association with a higher risk of bleeding in 102 patients with sepsis-driven new AF (OR: 0.96 [95% CI: 0.29–3.21) at 3 years [46]. However, their study included patients with acute coronary and pulmonary syndromes and thus may have been underpowered to detect a significant benefit specifically in the sepsis cohort. Furthermore, in both studies, the duration of anticoagulation was not explicitly mentioned and only a minority of patients received a DOAC. Therefore, in the acute stage of sepsis-induced AF, with limited evidence, and considering that sepsis may convey a physiological tendency towards bleeding [47], physicians may consider being cautious in fully anticoagulating patients.

What about anticoagulation in those at high-risk of ischaemic stroke in the sub-acute period after recovery from sepsis? The authors believe that sepsis-driven AF is an important clinical entity which, similarly to post-operative AF, should be taken seriously as it carries a high risk of recurrence in the medium to long term (around 50% based on the current data). Furthermore, the risk of ischaemic stroke remains elevated in long term. It is very likely that systemic changes as a result of sepsis unmask the pre-existing arrhythmogenic tendency of the atria and trigger AF. We suggest that after the acute stage of sepsis, in every patient with sepsis-driven AF, clinicians should carry out an assessment of the risk of ischaemic stroke using the CHA_2_DS_2_-VASc score and the risk of bleeding, and following careful consideration of the individual's views, offer anticoagulation. For those who are at a truly low risk of stroke or decide against anticoagulation, an option could be prolonged cardiac monitoring with close outpatient follow up and reconsideration of anticoagulation if AF recurs. As anticoagulating patients with sepsis-driven AF is currently outside of clear guidelines, integrated care that incorporates stroke prevention as well as management of other cardiovascular comorbidities is paramount, especially as it has shown to improve patient outcomes [48]. A truly holistic approach would incorporate the priorities set out in the ABC (Atrial fibrillation Better Care) pathway (‘*A*’ anticoagulation/avoid stroke; ‘*B*’ better symptom management; and ‘*C*’ cardiovascular risk and comorbidity risk reduction) [49] and this should be executed by coordinated and integrated pathways through primary and secondary care.

As yet, studies have not prospectively calculated the risk and benefit of starting anticoagulation in the population of patients who develop sepsis-driven AF, nor have they considered the optimum strategy or length of cardiac monitoring for these patients. Consequently, there is a clear need for pragmatically designed, prospective, randomised control trials to further asses the true risk of ischaemic stroke in patients with sepsis-driven AF, with the aim of understanding:

(1) The short-term recurrence rate of AF after sepsis.
(2) The short and long-term risks of ischaemic stroke in sepsis-driven AF.
(3) If there is a net-benefit from anticoagulation in these patients?
(4) When anticoagulation should be started? At the discovery of AF in hospital, or once the episode of sepsis is treated?

## 7 Conclusion

The data for sepsis-driven AF and the subsequent risk of ischaemic stroke and mortality are predominantly from retrospective studies conducted in heterogenous populations. Despite their limitations, we can elucidate that sepsis can cause AF, and in those with relevant risk factors with a cardiac substrate for AF, sepsis may well unmask pre-existing subclinical AF or cause new AF. In the short term, AF in the setting of sepsis increases hospital stays and the likelihood of inpatient stroke and mortality. Long-term data suggest that AF recurs in up to 50% of patients post-discharge, and an individual's risk of stroke remains raised for many years after the initial episode of sepsis. Some patients who develop sepsis are at increased risk of adverse effects such as bleeding in the short term and in the absence of good quality evidence, the risk of anticoagulation likely outweighs the benefits in the acute stage of sepsis. However, after the acute stage of sepsis, in every patient with sepsis-driven AF, clinicians should holistically assess the risk of ischaemic stroke and bleeding and educate their patient to make an informed decision regarding stroke prevention, which means anticoagulation in a number of high-risk patients.

## Funding

Dr Induruwa is funded by a NIHR Academic Clinical Lectureship (RG85316) and is supported by the British Heart Foundation Cambridge Centre of Research Excellence (RE/13/6/30180). Dr Warburton acknowledges funding support from the NIHR Eastern Clinical Research Network and the Cambridge Biomedical Research Centre (BRC).

## Ethical approval

None.

## Declaration of Competing Interest

Dr Khadjooi acknowledges travel grants from Bayer, Boehringer Ingelheim, Daiichi Sankyo and Pfizer.
